# Electrochemical Synthesis and Kinetic Evaluation of Electrooxidation of Acetaminophen in the Presence of Antidepressant Drugs

**Published:** 2015

**Authors:** Davood Nematollahi, Bahareh Feyzi Barnaji, Ameneh Amani

**Affiliations:** a *Faculty of Chemistry, University of Bu-Ali Sina, Hamedan, Iran.*; b *Payame Noor University (PNU), Hamedan, Iran.*; c *Department of Medicinal Plants Production, Nahavand University, Nahavand, Iran.*

**Keywords:** Acetaminophen, Paracetamol, Antidepressant drugs, Drug-drug interaction, Cyclic voltammetric

## Abstract

With the aim of obtaining information about drug-drug interaction (DDI) between acetaminophen and some of antidepressant drugs (fluoxetine, sertraline and nortriptyline), in the present work we studied the electrochemical oxidation of acetaminophen (paracetamol) in the presence of these drugs by means of cyclic voltammetry and Controlled-potential coulometry. The reaction between *N*-acetyl-*p*-benzoquinone-imine (NAPQI) produced from electrooxidation of acetaminophen and antidepressant drugs (see scheme 1) cause to reduce the concentration of NAPQI and decreases the effective concentration of antidepressants. The cyclic voltammetric data were analyzed by digital simulation to measure the homogeneous parameters for the suggesting electrode mechanism. The calculated observed homogeneous rate constants $\left(k_{\mathrm{obs}}\right)$ for the reaction of electrochemically generated *N*-acetyl-para benzoquinn-imine with antidepressant drugs was found to vary in the order $k_{\mathrm{obs}}^{\mathrm{nortriptyline}}$ > $k_{\mathrm{obs}}^{\mathrm{sertraline}}$ > $k_{\mathrm{obs}}^{\mathrm{fluxetine}}$ at biological pH.

## Introduction

**Figure 1:**
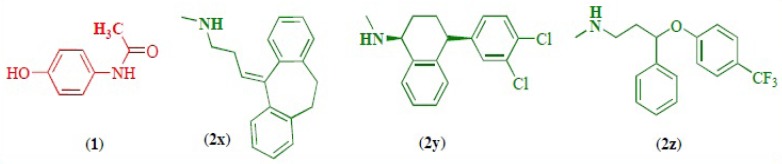
Chemical structure of acetaminophen (1), nortriptyline (2x), serteralin (2y) and fluxetine (2z).

As pharmaceutical technology continues to expand at a phenomenal rate, so does the incidence of drug interactions. A clinically relevant drug-drug interaction (DDI) occurs when the effectiveness or toxicity of one medication is altered by the administration of another medicine or a substance that is administered for medical purposes. Adverse consequences of DDIs may result from either diminished therapeutic effect or toxicity. The two drugs need not physically interact with each other to produce the effect. When the drug combination results in an undesired effect, the drug interaction becomes an adverse drug interaction. Drug interactions are much more common than adverse drug interactions (1-10). In one of the commonest treatment cases, acetaminophen (paracetamol) (1) is used with antidepressant drugs (2a-c) (Figure 1), so the study of the interaction between these drugs can be important both pharmaceutically and therapeutically.

It has been shown that *N-*acetyl-*p*-benzoquinone-imine (NAPQI) is the main *in-vivo *and *in-vitro *oxidation product of acetaminophen (11). Our previous studies show that the electrochemically generated NAPQI is a reactive intermediate and as a Michael acceptor, participates in different types of reactions (11-16). On the other hand, a literature survey shows that acetaminophen is metabolized by cytochrome P450 2E1 to the reactive metabolite NAPQI (11). Since the electrochemical oxidation parallels the cytochrome P450-catalysed oxidation in liver microsomes, it was interesting to study the anodic oxidation of acetaminophen in the presence of antidepressant drugs (2x-z). In this work we would like to report a simple electrochemical procedure on the drug-drug interaction between acetaminophen and antidepressant drugs (Figure 1) and synthesis a new product of electro oxidation of acetaminophen in the presence of nortryptline (2x) at biological pH. Moreover the observed homogeneous rate constants $\left(k_{\mathrm{obs}}\right)$ of the reaction of NAPQI with antidepressant drugs (Figure 1, 2x-z) have been estimated by digital simulation of cyclic voltammograms.

## Experimental

Apparatus

Cyclic voltammetry was performed using a SAMA500 potentiostat/galvanostat. Controlled-potential coulometry was performed using a potentiostat/galvanostat system model BHP 2061-C. The working electrode used in the voltammetry experiments was a glassy carbon disc (1.8 mm^2^ area) and a platinum wire was used as the counter electrode. The working electrode used in controlled-potential coulometry and macroscale electrolysis was an assembly of four carbon rods (31 cm^2^) and large platinum gauze constituted the counter electrode. The working electrode potentials were measured versus SCE (all electrodes from AZAR Electrode Co. Iran). Experimental conditions were as reported in our earlier paper (17). The homogeneous rate constants were estimated by analysing the cyclic voltammetric responses using cyclic voltammogram digital simulation software (DigiElch SB) version 2.0 (18).

*Reagents*

Acetaminophen (1) was reagent grade material from Aldrich. nortriptyline (2x) and serteralin (2y) were obtained from Rouz Darou Pharmaceutical Co. and fluxetine (2z) was obtained from Jalinous Pharmaceutical CO. All of solvents were of pro-analysis grade from E. Merck. These chemicals were used without further purification.

*Electroorganic Synthesis of 6*

In a typical procedure, 60 mL of phosphate buffer solution (0.2 M, pH 7.0) in water/acetonitrile (70/30), containing acetaminophen (1) (0.5 mmol) and nortriptyline (2a) (0.5 mmol) was electrolyzed at 0.30 V versus the SCE in a two-compartment cell. The electrolysis was terminated when the current decayed to 5% of its original value. At the end of electrolysis the precipitated solid was collected by filtration and was washed several times with water. After washing, product was characterized by IR, and MS. 

*Characterization data*

Mp. 250-252 ^◦^C (Dec.). IR (KBr): 3435.8, 3016.7, 2769.6, 2426.9, 1613.8, 1485.5, 1384.4, 1161.72, 1029.5, 777.56, 756.4, 594.9 cm^-^^1^ MS: *m/z *(relative intensity) = 495 (M, 3), 494 (6), 369 (6), 319 (5), 263 (21), 219 (64), 202 (100), 178 (40), 152 (61), 91 (50), 57 (74).

## Results and Discussion

*Voltammetric studies*

 Cyclic voltammogram of 1.0 mM of acetaminophen (1) in aqueous solution containing 0.2 M phosphate buffer at pH 7 shows one anodic (A_1_) and corresponding cathodic peak (C_1_), corresponding to the transformation of acetaminophen to *N-*acetyl-*p-*benzoquinone-imine (NAPQI) and *vice versa* within a quasi-reversible tow-electron process (Figure 2, curve a). (13-15) A peak current ratio $\left(\frac{l_{p}^{\mathrm{c1}}}{l_{p}^{\mathrm{A1}}}\right)$ of nearly unity particularly during the recycling of potential can be considered as criteria for the stability of *NAPQI* produced at the surface of electrode under the experimental conditions (14).

**Figure:**
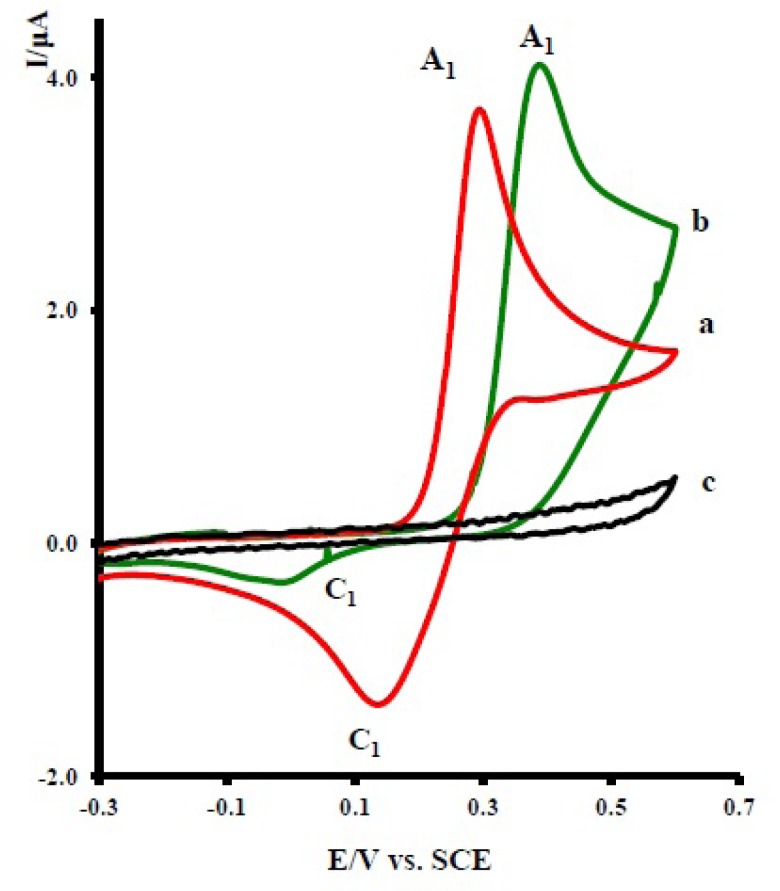
Cyclic voltammograms of 1.0 mM acetaminophen (1): (a) in the absence, (b) in the presence of 10.0 mM nortriptyline (2x), and (c) 10.0 mM nortriptyline (2x) in the absence of acetaminophen at a glassy carbon electrode in water/acetonitrile (70/30) solution containing 0.2 M phosphate buffer (pH = 7.0). Scan rate: 10 mVs^-1^, *T* = 25 ± 1ºC

The oxidation of 1 in the presence of 10 mM of nortriptyline (2x) was studied in some detail. Figure 2 curve b, shows the cyclic voltammogram of 1 in the presence of 2x. The voltammogram exhibits one anodic peak (A_1_) and its cathodic counterpart (C_1_). The comparison of peak C_1_ in the absence and presence of 2x shows a decrease in the current for the later. The observed shift of the A_1_ peak in curve b, relative to curve a, is probably due to the formation of a thin film of product at the surface of the electrode, inhibiting to a certain extent the performance of the electrode process. In this figure, curve c is the voltammogram of 2x in the same condition and in the absence of 1.

**Figure 3.**
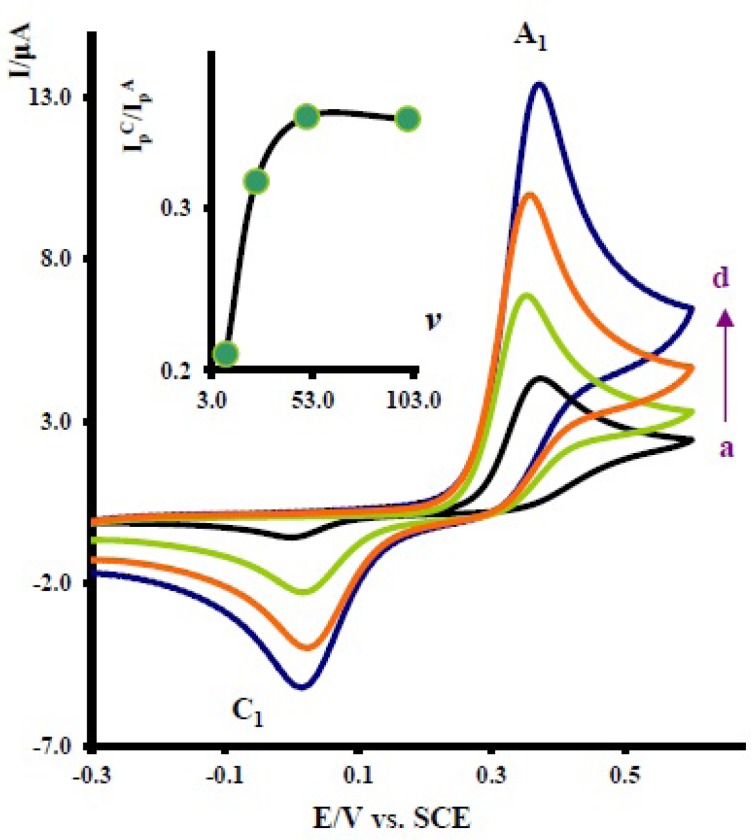
Cyclic voltammograms of 1 mM acetaminophen in the presence of 10.0 mM nortriptyline at a glassy carbon electrode in water/acetonitrile (70/30) solution containing 0.2 M phosphate buffer (pH = 7.0) at various scan rates. Scan rates from a to d are: 10, 25, 50 and 100 mVs^-1^. Inset: variation of peak current ratio $\left(\frac{l_{p}^{\mathrm{c1}}}{l_{p}^{\mathrm{A1}}}\right)$*vs* scan rate

**Figure 4:**
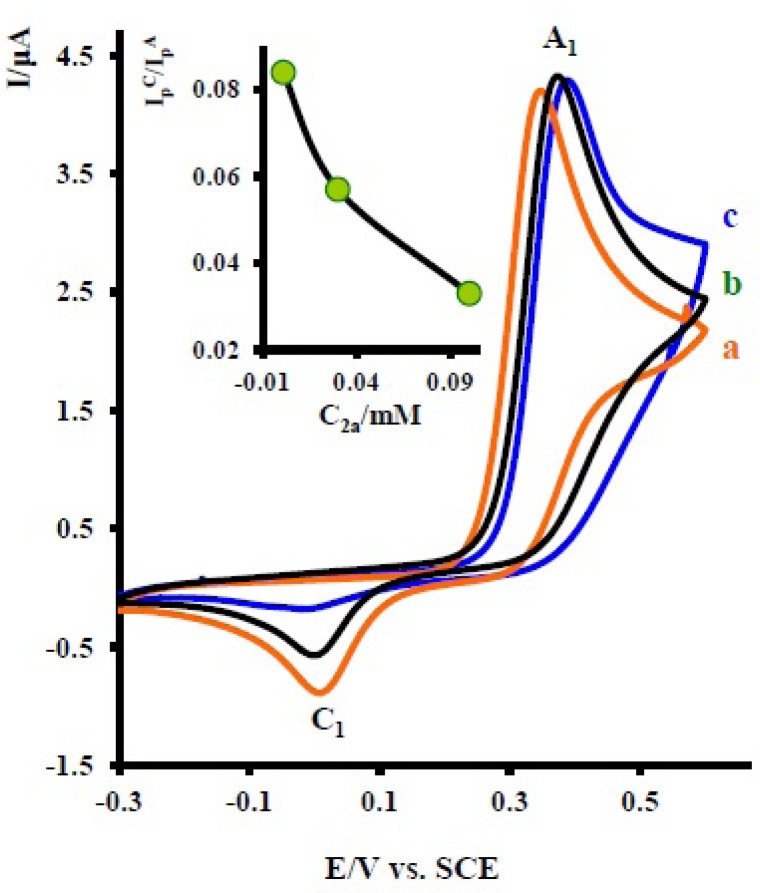
Cyclic voltammograms of 1 mM acetaminophen (1) in the presence various concentration of nortriptyline (2x) at a glassy carbon electrode in water/acetonitrile (70/30) solution containing 0.2 M phosphate buffer (pH = 7.0) at 10 mVs^-1^ scan rate. Concentrations from a to c are: 0.1, 0.01 and 0.03 mM. Inset: variation of peak current ratio $\left(\frac{l_{p}^{\mathrm{c1}}}{l_{p}^{\mathrm{A1}}}\right)$*vs* concentration ratio (nortriptyline (2x) / acetaminophen (1)). *T* = 25 ± 1 ºC.

The effect of scan rate on the electrochemical behavior of 1 in the presence of 2x was also studied. Figure 3 shows the typical cyclic voltammograms obtained for 1 mM of 1 in the presence of 10 mM nortriptyline (2x) at various potential scan rates. As can be seen, with increasing scan rate, the peak current ratio, $\left(\frac{l_{p}^{\mathrm{c1}}}{l_{p}^{\mathrm{A1}}}\right)$, increases (Figure 3 Inset). To gain further information, the electrochemical oxidation of acetaminophen (1) was studied in various concentrations of 2x. Figure 4 shows the cyclic voltammograms of 1 at various concentrations of 2x. As can be seen the peak current ratio $\left(\frac{l_{p}^{\mathrm{c1}}}{l_{p}^{\mathrm{A1}}}\right)$decrease with increasing of the concentration of 2x. This implies that the chemical reaction (DDI) occurred after the electron transfer process (19). 

The existence of a subsequent chemical reaction between NAPQI and 2x is supported by the following evidences: (a) decrease in $\left(l_{p}^{\mathrm{c1}}\right)$ during the reverse scan, which could be indicative of the fact that electrochemically generated NAPQI is removed by chemical reaction with 2x. (b) Dependency of the peak current ratio $\left(\frac{l_{p}^{\mathrm{c1}}}{l_{p}^{\mathrm{A1}}}\right)$on the potential sweep rate (Figure 3). In this case, for the highest sweep rate employed, a well-defined cathodic peak C_1_ is observed. For lower sweep rates, $\left(\frac{l_{p}^{\mathrm{c1}}}{l_{p}^{\mathrm{A1}}}\right)$ become less than one and increases with increasing scan rate (Figure 3, inset). This is indicative of departure from the intermediate and arrival to the diffusion region with increasing sweep rate (19). (c) Dependency of $\left(\frac{l_{p}^{\mathrm{c1}}}{l_{p}^{\mathrm{A1}}}\right)$on the concentration of 2x. This is related to the increase of the homogeneous reaction rate of the following chemical reaction. (d) Increasing of $\left(l_{p}^{\mathrm{A1}}\right)$ in the presence of 2x (Figure 2 curve b). This can be related to the increase of apparent number of electrons from two to six (see coulometry section).

**Figure 5:**
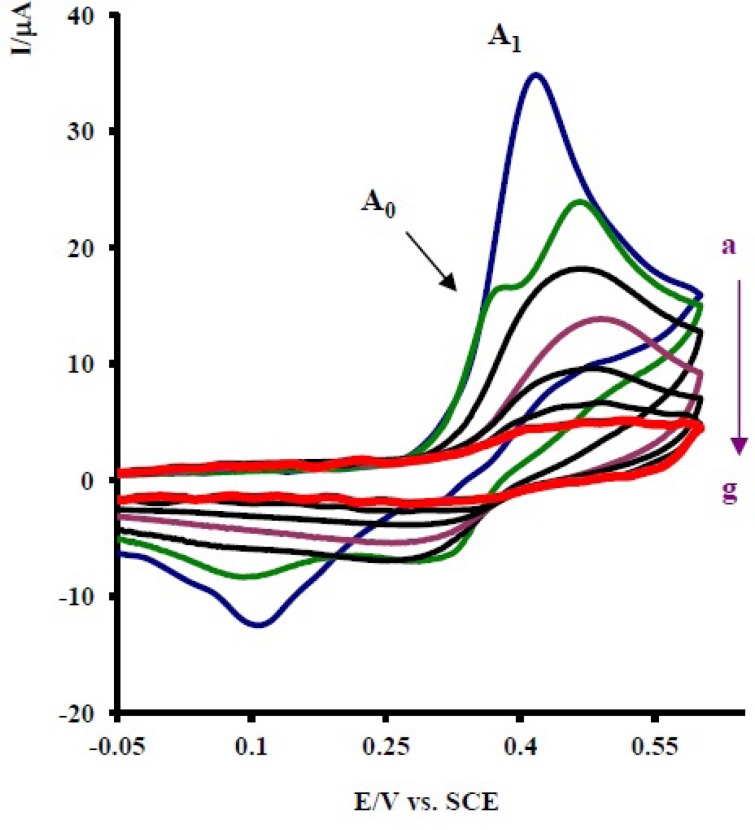
Cycilc voltammograms of 0.1 mmol acetaminophen (1) in the presence of 0.1 mmol nortriptyline (2x), in 0.2 M phosphate buffer, pH 7, during controlled potential coulometry at 0.30 V *vs. *SCE, after consumption of: (a) 0, (b) 10, (c) 20, (d) 30, (e) 40, (f) 50 and (g) 55 C. Scan rate: 100 mV s ^-1^. Inset: variation of peak current *vs *charge consumed. *T* = 25 ± 1 °C

**Scheme. 1:**
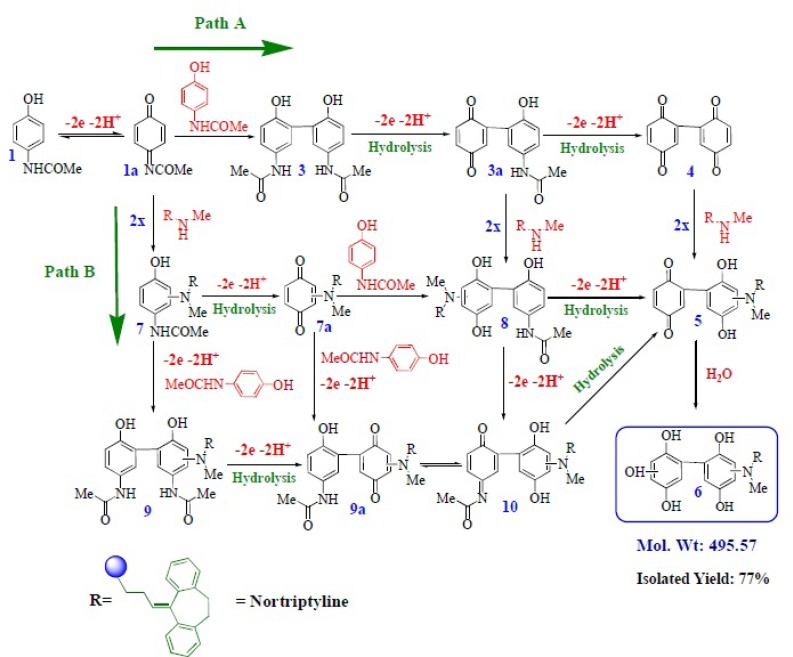
Proposed mechanisms for the electrochemical oxidation of acetaminophen (1) in the presence of 2x-z

Controlled-potential coulometry was performed in aqueous solution containing 0.1 mmol of 1 and 0.1 mmol of 2x at 0.30 V *versus *saturated calomel electrode (SCE). The electrolysis progress was monitored using cyclic voltammetry (Figure 5). It is shown that, proportional to the advancement of coulometry, the anodic peak A_1_ decreases and a new anodic peak (A_0_) appears. The anodic peak A_0_ is probably related to the oxidation of an intermediate that is produced in the electrolysis cell (7a or 9a in Scheme 1). Anodic and cathodic peakas (A_1_/C_1_ and A_0_/C_0_) disappear when the charge consumption becomes about 6e^-^ per molecule of 1.

Electrochemical and spectroscopic data in accompanied by previously published data (12-15) allow us to propose the mechanism presented in Scheme 1 for the electrooxidation of acetaminophen (1) in the presence of 2x-z. It seems that because of the good reactivity (decrease in $l_{p}^{\mathrm{c1}}$ during the reverse scan) between NAPQI and 2x at the surface electrode in time window of cyclic voltammetry (10^-7^-1 s), so we can expect that Michael addition reaction takes place prior to the hydrolysis reaction in controlled-potential coulometry (a method with time windows, 100–3000 s (19)) according to the path B (Scheme 1) leading to the product 6. Since the oxidation of 6 occurs at more positive potentials, the overoxidation of 6 was circumvented during the controlled potential preparative reaction. The electroorganic synthesis of 6 has been performed using oxidation of 1 in the presence of 2x as described in the experimental section. According to our results, the anodic peak of the voltammograms presented in Figure 2 (A_1_) pertains to the oxidation of acetaminophen (1) to the *N-*acetyl-*p*-benzoquinone-imine (NAPQI) 1a. Obviously, the cathodic peak C_1_ is corresponding to the reduction of *N-*acetyl-*p*-benzoquinone-imine (NAPQI) 1a.

**Figure 6.**
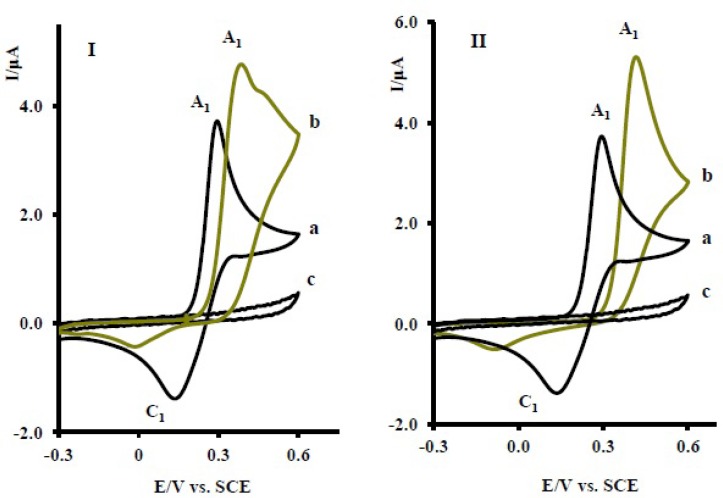
(I) Cyclic voltammograms of 1 mM acetaminophen (1) in the absence (a), presence (b) of 10 mM fluxetin (2z) and (c) 10 mM fluxetin **(2z**) (II) Cyclic voltammograms of 1 mM acetaminophen (1) in the absence (a), presence (b) of 10 mM serteralin (2y) and (c) 10 mM serteralin (2y) at the glassy carbon electrode in 0.2 M phosphate buffer, pH 7 at scan rate of 10 mVs^-1^, *T* = 25 ± 1ºC

The proposed mechanism was established by using Ms and IR technique. In the mass spectrum of compound 6, molecular ion [M + 1H] was recorded. This mass is related to protonation of the nortriptyline (2x) group (20-22) and is another proof for production of 6 in electrooxidation of 1 in the presence of 2x. The electrochemical oxidation of acetaminophen (1) in the presence of sertraline (2y) and fluoxetine (2z) was also investigated (Figure 6). The same behavior has been seen for the electrochemical oxidation of 1 in the presence of 2y, z.

*Simulation*

Electrochemical oxidation of acetaminophen (1) in various pHs was tested by digital simulation. The simulation was carried out assuming semi-infinite one-dimensional diffusion and planar electrode geometry (14). The experimental parameters entered for digital simulation consisted of the following: *E*_start_, *E*_switch_, *E*_end_, *t*=25^◦^^C^ and analytical concentration of acetaminophen (1). The transfer coefficient (*α*) was assumed to be 0.5 and the formal potentials were obtained experimentally as the mid point potential between the anodic and cathodic peaks (*E*_mid_). The heterogeneous rate constant (0.002cms^−^^1^) for oxidation of acetaminophen (1) was estimated by use of an experimental working curve (14,23). All these parameters were kept constant through out the fitting of the digitally simulated voltammogram to the experimental data. The parameter $k_{\mathrm{obs}}$ was allowed to change through the fitting processes. It should be noted that since in the absence of 2x-z, the peak current ratio $\left(\frac{l_{p}^{\mathrm{c1}}}{l_{p}^{\mathrm{A1}}}\right)$of 1 because of the participation of NAPQI (1a) in side reaction(s), is less than unity, the homogeneous rate constant of side reactions must be calculated, in the absence of nucleophile (2x-z), firstly, then *k*_obs_ for the reaction of 1a with 2x-z estimated in the next step according to simplified Scheme 1. Table 1 shows the $k_{\mathrm{obs}}$ as a function of pH. As shown in Table 1, $k_{\mathrm{obs}}$ is strongly dependent to solution’s pH and type of antidepressant and increase with increasing of pH. The observed trend is expected, because of the more deprotonation of donating atom(s) in the structures of 2x-z and more activation of them towards Michael addition reactions with NAPQI (24). Moreover $k_{\mathrm{obs}}$ for these drugs varied in the order of $k_{\mathrm{obs}}^{\mathrm{nortriptyline}}$ > $k_{\mathrm{obs}}^{\mathrm{sertraline}}$ > $k_{\mathrm{obs}}^{\mathrm{fluxetine}}$. This can be due to the absence of any electron withdrawing group (EWG) (Cl, CF_3_) in the structure of 2x (Figure 1) (13). 

**Figure 7:**
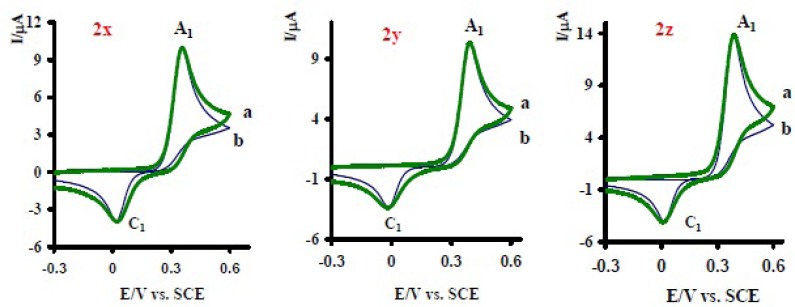
Cyclic voltammogrms of 1mM acetaminophen (1) and 10mM antidepressant drugs: nortriptyline (2x), serteralin (2y) and fluxetine (2z) at pH= 7.4, (a) experimental (b) simulated. Scan rate 70 mVs^-1^

**Table 1.** Observed homogeneous rate costants, *k*_obs_(s^-1^), for the studied acetaminophen in the presence of nortriptyline (2x), sertraline (2y) and fluoxetine (2z).

pH	k_obs_^a^ of 1a with 2x	k_obs_^a^ of 1a with 2y	k_obs_^a^ of 1a with 2z
**2.9**	7.61±64	5.45±0.16	1.35±0.07
**4**	9.40±1.02	7.1±0.44	1.95±0.06
**4.8**	11.43±0.97	8.66±1.52	3.84±0.22
**7.5**	14.00±0.84	12.13±1.53	7.04±0.25
**8.7**	20.32±2.18	18.00±1.73	11.03±1.65
**9.7**	44.00±3.87	33.5±3.53	21.53±3.13

a Each second-order rate coefficient is the average of five determinations and is reported together with the standard deviation.

## Conclusions

This work presents sound electrochemical data for the reaction of *N-*acetyl-*p-*benzoquinone-imine (NAPQI) derived from the oxidation of acetaminophen (1) with antidepressant drugs (2x-z). This reaction (see scheme 1), reduces the concentration of NAPQI and decreases the effective concentration of antidepressants. In addition, this work introduces electrochemistry as a *"powerful tool"* to accomplish diagnostic tests in medical and pharmaceutical sciences (25-28) and the synthesis of new organic compounds.
